# Role of LptD in Resistance to Glutaraldehyde and Pathogenicity in *Riemerella anatipestifer*

**DOI:** 10.3389/fmicb.2019.01443

**Published:** 2019-06-21

**Authors:** Li Huang, Mingshu Wang, Ting Mo, Mafeng Liu, Francis Biville, Dekang Zhu, Renyong Jia, Shun Chen, Xinxin Zhao, Qiao Yang, Ying Wu, Shaqiu Zhang, Juan Huang, Bin Tian, Yunya Liu, Ling Zhang, Yanling Yu, Leichang Pan, Mujeeb Ur Rehman, Xiaoyue Chen, Anchun Cheng

**Affiliations:** ^1^Institute of Preventive Veterinary Medicine, Sichuan Agricultural University, Chengdu, China; ^2^Research Center of Avian Disease, College of Veterinary Medicine, Sichuan Agricultural University, Chengdu, China; ^3^Key Laboratory of Animal Disease and Human Health of Sichuan Province, Chengdu, China; ^4^Pasteur Institute, Paris, France

**Keywords:** *Riemerella anatipestifer*, lipopolysaccharide, LptD, hydrophobic drug resistance, membrane permeability

## Abstract

*Riemerella anatipestifer* is a gram-negative bacterium that causes disease in ducks and other birds. Despite being an important pathogen in poultry, the pathogenesis and drug resistance mechanisms of this bacterium are poorly understood. An analysis of our unpublished RNA-Seq data showed that *lptD*, a gene encoding one of the lipopolysaccharide transport components, is transcribed at higher levels in strain CH-1 than in strain ATCC11845. In addition, strain CH-1 has been shown to display broader drug resistance than strain ATCC11845. Since LptD is involved in LPS biogenesis and drug resistance, we wondered if *lptD* is associated with increased *R. anatipestifer* resistance to glutaraldehyde, a disinfectant used in the production industry. In this study, the minimal inhibitory concentration (MIC) of glutaraldehyde for strain CH-1 was determined to be 0.125% (vol/vol), whereas an MIC of 0.05% (vol/vol) was observed for strain ATCC11845. Furthermore, the level of *lptD* transcription in strain CH-1 was consistently 2-fold higher than that observed in strain ATCC11845. Moreover, *lptD* transcription was upregulated in both strains at a subinhibitory concentration of glutaraldehyde. The role of *lptD* in *R. anatipestifer* was further assessed by constructing an ATCC11845 mutant strain with low *lptD* expression, *R. anatipestifer* ATCC11845 *lptD*^−^. The growth of *R. anatipestifer* ATCC11845 *lptD*^−^ was severely impaired, and this strain was more susceptible than the wild-type strain to glutaraldehyde. Moreover, compared to the wild-type strain, *R. anatipestifer* ATCC11845 *lptD*^−^ exhibited decreased biofilm formation and was more sensitive to duck serum. Finally, low *lptD* expression led to decreased colonization in ducklings. These results suggest that LptD is involved in *R. anatipestifer* glutaraldehyde resistance and pathogenicity.

## Introduction

The cell envelope of gram-negative bacteria includes an inner membrane (IM), a periplasm, and an outer membrane (OM) (Ruiz et al., 2006). The OM is an asymmetric bilayer with inner and outer leaflets composed of phospholipids and lipopolysaccharide (LPS), respectively (Kamio and Nikaido, 1976). LPS forms a barrier to protect bacteria from hydrophobic antibiotics, dyes and detergents (Nikaido, 2003). Using *Escherichia coli* as an model system, the biogenesis of LPS has been shown to be a three-step process involving its synthesis in the cytoplasm, transport across the IM to the periplasmic space and insertion into the outer leaf of the OM (Raetz and Whitfield, 2002). The machinery that mediates LPS transports across the IM to the periplasmic space and its insertion into the OM has been well characterized through intense research during the last decade (Putker et al., 2015). Seven LPS transport proteins (Lpt), LptA, LptB, LptC, LptD, LptE, LptF, and LptG, have been reported to be involved in the transport of LPS in *E. coli* (Sperandeo et al., 2009; Putker et al., 2015). Furthermore, these proteins have been shown to localize to three different regions in the cell envelope. LptB, LptF, and LptG form an ABC transporter in the IM that provides the energy for LPS detachment (not flipping) from the IM and transport across the periplasm (Narita and Tokuda, 2009). LptA and LptC form a periplasmic complex that connects with LptD/E and LptBFG (Freinkman et al., 2012). LptD and LptE form a hetero-oligomeric complex in the OM (Wu et al., 2006) that is responsible for the translocation of LPS to the OM and its final assembly on the cell surface (Chng et al., 2010b). Decreased *lptD* transcription causes protein extravasation and membrane protein mislocalization in *E. coli*, suggesting that LptD is essential in this bacterial species and promotes correct cell membrane assembly (Braun and Silhavy, 2002). However, LptD is non-essential in *Neisseria meningitidis*, as bacteria are viable without LPS (Steeghs et al., 1998). Furthermore, LptD has been shown to be involved in organic solvent tolerance in *E. coli* and *Helicobacter pylori* (Ohtsu et al., 2004; Chiu et al., 2009).

*Riemerella anatipestifer* is a gram-negative bacterium that belongs to the *Flavobacteriaceae* family and causes septicemic diseases in ducks, geese, turkeys, and other birds (Segers et al., 1993). At present, because significant cross-protection has not been observed for these 21 different serotypes (Pathanasophon et al., 1995, 2002), it is difficult to control this disease in the duck production using vaccines. A number of factors have been reported to be involved in the pathogenesis of *R. anatipestifer* (Chang et al., 1998; Crasta et al., 2002; Hu et al., 2011; Wang et al., 2017; Yi et al., 2017; Liu et al., 2018). The wide use of antibiotics during poultry feeding has promoted the emergence of *R. anatipestifer* strains that are resistant to multiple antibiotics (Zhong et al., 2009; Luo et al., 2015, 2018; Huang et al., 2017; Zhang et al., 2017; Zhu et al., 2018). In a previous study, we showed that the strain CH-1 is resistant to many antibiotics, with the strain ATCC11845 being more susceptible to the tested antibiotics than CH-1 (Luo et al., 2015; Xing et al., 2015). The resistance of strain CH-1 and strain ATCC11845 to organic solvents is currently unknown, and glutaraldehyde is a commonly used disinfectant in poultry. According to our unpublished RNA-Seq data, *lptD* is transcribed at higher levels in strain CH-1 than that in strain ATCC11845 (Supplementary Figure S1 and Supplementary Tables S1, S2). In this study, we investigated whether strain CH-1 is more resistant to glutaraldehyde than strain ATCC11845 and if this phenotype is associated with the level of *lptD* transcription in these strains, the results of which will be helpful for laying a foundation for studying resistance mechanisms in *R. anatipestifer*.

## Materials and Methods

### Bacterial Strains, Primers and Growth Conditions

The bacterial strains and primers used in this study are shown in Table 1. *R. anatipestifer* was grown in GC broth (GCB) or tryptone soy broth (TSB) medium at 37°C with shaking (Liu et al., 2017). GCB agar plates were prepared by supplementing GCB with 1.5% agar. Alternatively, *R. anatipestifer* strains were also grown on LB agar supplemented with 5% sheep blood. When required, media were supplemented with erythromycin at a final concentration of 1 μg/ml or with different concentrations of glutaraldehyde or sodium dodecyl sulfate (SDS).

**Table 1:** Strains and primers used in this study.

*R. anatipestifer* strains	Genotype or description	Source or reference
CH-1	CH-1, Kanamycin resistance	Laboratory collection
ATCC11845	ATCC11845, Kanamycin resistance	Laboratory collection
ATCC11845 *lptD*^−^	ATCC11845 with low expression of *lptD*, Kanamycin resistance	This study

**Primers**	**Sequence**	**Organism**

lptD Pro upP1	AAACATAATTAAGCCCTTTCAAAGCAGGATCTCCCTCAT	ATCC11845
lptD Pro upP2	GGAAAGTGGTTATTGAAAATTTGGCTTCAAAATTAGT	ATCC11845
ErmP1	AAATTTTCAATAACCACTTTCCAGTCTTACGAAGCACGAACCCCCTGC	CH-1
ErmP2	TTGGCTTCAACGACTTTGAACTACGAAGGATGAAATTTTTCAGGG	CH-1
lptD Pro downP1	TCCTTCGTAGTTCAAAGTCGTTGAAGCCAAATTTT	ATCC11845
lptD Pro downP2	TTATAGCCTAGTTCAGGGCGAATGTTCCAGCTTCCTTTGG	ATCC11845
qrecA P1	TGAAACTAGGTGATGGTACG	ATCC11845
qrecA P2	GGGTAGGTGGTTATCCTAAG	ATCC11845
qlptD P1	CCTCGTAAAGAATCCCTCGAG	ATCC11845
qlptD P2	CCCAGTTTATGGATATGTAATCTGC	ATCC11845
qRA0C_1120 P1	AGGGCTATCAAAGATTCTGGCG	ATCC11845
qRA0C_1120 P2	CTTTGTGCAAGGGCCAGATC	ATCC11845
qRA0C_1122 P1	CAACCAATAACGCTCCTGCTG	ATCC11845
qRA0C_1122 P2	TCTATTCCTGTAACCAATTCGCC	ATCC11845

### Construction of an *R. anatipestifer* ATCC11845 Strain Expressing Low Levels of *lptD*

An *R. anatipestifer* ATCC11845 strain expressing low levels of *lptD* was constructed using the natural transformation method as described previously (Liu et al., 2017). Briefly, ∼800-bp fragments upstream and downstream of the start codon of the *lptD* gene were amplified using the primer pairs lptD Pro-upP1/lptD Pro-upP2 and lptD Pro-downP1/lptD Pro-downP2, respectively (Table 1). A 994-bp erythromycin resistance cassette with a promoter was amplified from strain CH-1 (Luo et al., 2015) using the primers ErmP1/ErmP2 (Table 1). The three PCR fragments were fused by the overlap PCR method (Xiong et al., 2006), purified using a Universal DNA Purification kit (TIANGEN, Beijing, China) and served as donor DNA. Wild-type strain ATCC11845 served as the recipient strain for the fused fragments, which were introduced by natural transformation. Transformants in which the erythromycin resistance cassette with a promoter was inserted upstream of the *lptD* start codon were selected for on LB plates supplemented with 5% sheep blood and 1 μg/ml erythromycin. A strain expressing low levels of *lptD*, strain ATCC11845 *lptD*^−^, was verified by PCR by amplifying the erythromycin resistance cassette using the primers ErmP1/ErmP2 (Table 1).

### Growth Rate Determination

The *in vitro* growth rates of the strains were determined as described previously (Wang et al., 2017). Briefly, the bacterial cells were grown overnight on LB plates supplemented with 5% sheep blood, after which a single colony was inoculated into 5 ml of TSB and cultured at 37°C with agitation for 10 h. Subsequently, the cultures were adjusted to an OD_600_ of 0.05 in 20 ml of fresh and grown at 37°C with shaking at 180 rpm, with OD_600_ values determined at every 2 h for 16 h.

### Determination of the Minimal Inhibitory Concentrations (MICs)

The MICs of glutaraldehyde, SDS and antibiotics (novobiocin, imipenem rifampicin and polymyxin B) for *R. anatipestifer* were determined in 96-well microtiter plates as described in a previous study (Huang et al., 2017). Briefly, after culturing the strains to the logarithmic growth phase, the turbidity of the cultures was adjusted to 10^7^ colony-forming units (CFU)/ml (100 μl/well). A culture without antibiotics was included as positive control, and a sample of uninoculated broth was used as a negative control. The experiments were repeated three times, with the results determined after a 24 h incubation at 37°C.

### Biofilm Formation Assays

The *R. anatipestifer* strains were for biofilm formation in tubes as described previously with slight modifications (Kita et al., 2016). Cells of the *R. anatipestifer* strains were collected from LB agar plates supplemented with 5% sheep blood and resuspended in phosphate-buffered saline (PBS). The cells were washed three times with PBS. The bacterial suspensions were adjusted to an OD_600_ of 1 and then were inoculated into 5 ml of TSB supplemented with 5% serum at an OD_600_ of 0.1 in glass tubes and cultured at 37°C without shaking. After incubating for 24 h, the OD_600_ values of the cultures was determined, and the contents of each tube was carefully removed with a pipette. The tubes were washed three times with PBS and stained with 0.1% crystal violet for 30 min at room temperature. After removing the crystal violet solution and washing each tube twice with PBS, the biomass-associated crystal violet was extracted with 3 ml of absolute ethyl alcohol, and the absorbance at OD_580_ was measured.

**Table 2:** MICs [μg/ml or % (vol/vol)] of various antimicrobial agents for *R. anatipestifer* strains.

Strain	Glutaraldehyde	SDS	Imipenem	Rifampicin	Polymyxin B	Novobiocin
CH-1	0.125	ND	ND	ND	ND	ND
ATCC11845	0.05	0.00125	0.5	0.025	>500	0.05
ATCC11845 *lptD*^−^	0.0125	0.00125	0.125	0.005	250	0.025

*ND, not determined.*

### Serum Bactericidal Assay

Serum lacking antibodies to *R. anatipestifer* was obtained from non-immune ducks and filter-sterilized (0.22 μm) for bactericidal assays. Briefly, after adjusting cultures of each bacterial strain tested to an OD_600_ of 1, the serum was added to the cell cultures at final concentrations of 10 or 20%. Cell cultures without serum were used as a negative control. The samples in group were incubated for 15 and 30 min at 37°C. Subsequently, the cell cultures were serially diluted and plated onto LB agar supplemented with 5% sheep blood. The survival rate was calculated as CFU/ml in pooled serum divided by the CFU/ml of the negative control.

### Quantitative Real-Time PCR

Quantitative real-time PCR (qRT-PCR) was performed as described in a previous study with some modifications (Liu et al., 2016). Briefly, the tested strains were cultured in 20 ml of TSB to the exponential growth phase, after which RNA was extracted from the cell cultures at 1 OD600 using an RNAprep pure Cell/Bacteria kit (TIANGEN^TM^, Beijing, China). cDNA was generated using HiScript reverse transcriptase according to the manufacturer’s instructions. qRT-PCR was performed to determine the transcript level of *lptD* using SYBR Green Master Mix (Vazyme: Q111-01) and the primers qlptD P1/qlptD P2 (Table 1). The gene *recA* served as an internal reference gene to normalize the level of *lptD* expression. Three samples and technical replicates were performed, and the fold change was calculated using the ΔΔCt method as previously described (Pfaffl, 2001).

### Colonization Assays

Colonization studies were conducted using ten 3-day-old Pekin ducklings per group. The wild-type strain and the mutant expressing low levels of *lptD* were cultured overnight on sheep blood plates at 37°C. Subsequently, bacterial cells were scraped from the plates, resuspended in TSB medium and cultured to the exponential phase at 37°C with shaking at 180 rpm. After collecting the bacteria by centrifugation at 4°C for 10 min, the cells were washed three times and suspended in PBS. Subsequently, 10^9^ CFU of the bacterial suspensions were intramuscularly injected into the legs of ducklings. The blood, livers and brains of the ducklings were collected at 12 and 18 h postinoculation and homogenized in PBS (0.1 g sample/0.9 ml PBS) using a Nasco WHIRL-PAK (B01245WA, United States) as previously described (Liu et al., 2018). The homogenized contents were serially diluted and spread onto blood agar plates for enumeration.

### Ethics Statement

All ducks were handled in strict adherence to the recommendations of the local animal welfare bodies and the Sichuan Agricultural University Ethics Committee (SYXK2014-187). The protocol was approved by the Sichuan Agricultural University Ethics Committee.

### Statistical Analysis

Statistical analysis was performed using GraphPad Prism 7.0 for Windows (GraphPad Software Inc., San Diego, CA, United States). The significance of the data was ascertained using Student’s *t*-test, and a value of *P* < 0.05 was considered significant.

## Results

### *R. anatipestifer* ATCC11845 Is More Susceptible to Glutaraldehyde Than Strain CH-1

Glutaraldehyde is both an organic solvent and a hydrophobic drug that is commonly used as a disinfectant (Chiu et al., 2009). To explore whether different *R. anatipestifer* strains have different tolerances to glutaraldehyde, the MICs of glutaraldehyde for strain CH-1 and strain ATCC11845 were determined. The results showed that the MIC of glutaraldehyde for strain CH-1 and strain ATCC11845 was 0.125% (vol/vol) and 0.05% (vol/vol), respectively (Table 2), revealing that strain ATCC11845 is more susceptible to glutaraldehyde than strain CH-1.

### *lptD* Is Transcribed at Lower Levels in *R. anatipestifer* ATCC11845 Than Strain CH-1

**FIGURE 1:**
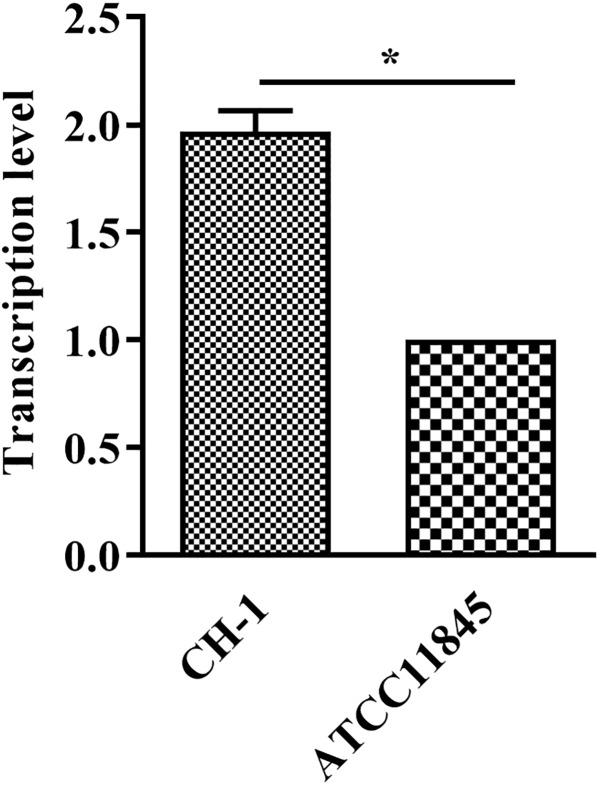
Transcription of *lptD* in *R. anatipestifer* CH-1 and ATCC11845. RNA was extracted and performed qRT-PCR. The data were analyzed using Student’s *t*-test. The error bars represent the standard deviations of three independent experiments, ^∗^, *p* < 0.05.

Bacterial resistance to glutaraldehyde has been reported to be associated with *lptD* (Chiu et al., 2009). Inprevious study, RNA-Seq data showed that *lptD* had higher transcription level in strain CH-1, compared to that in strain ATCC11845 (Supplementary Figure S1 and Supplementary Tables S1, S2). To verify whether the tolerance of different *R. anatipestifer* strains to glutaraldehyde is caused by *lptD*, the transcription of *lptD* in strain CH-1 and strain ATCC11845 was assessed by qRT-PCR as described in a previous study (Chiu et al., 2009). As shown in Figure 1, the level of *lptD* transcription in strain CH-1 was 2-fold higher than that observed in strain ATCC11845. Thus, the different tolerance of the strains to glutaraldehyde was predicted to be associated with the level of *lptD* transcription in *R. anatipestifer*.

### Transcription of the *lptD* Gene Is Induced by Glutaraldehyde in *R. anatipestifer* CH-1 and ATCC11845

**FIGURE 2:**
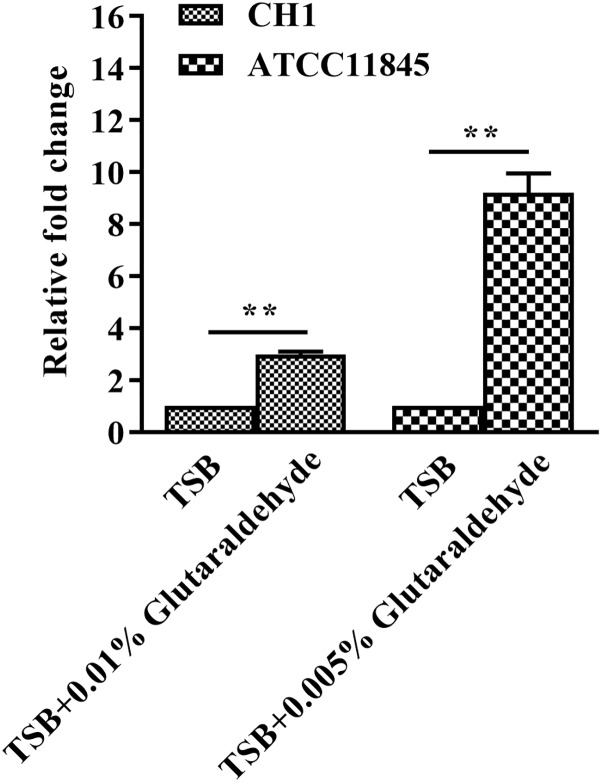
Changes in *lptD* transcription in *R. anatipestifer* CH-1 and ATCC11845 in TSB and TSB with glutaraldehyde. qRT-PCR analysis of the relative expression of *lptD* in strain CH-1 and strain ATCC11845 in TSB and TSB with glutaraldehyde (0.01% (vol/vol) and 0.005% (vol/vol) for strain CH-1 and strain ATCC11845, respectively).The data were analyzed using Student’s *t*-test. The error bars represent the standard deviations of three independent experiments, ^∗∗^, *p* < 0.01.

To further assess whether the tolerance of *R. anatipestifer* to glutaraldehyde is correlated with *lptD* expression, strain CH-1 and strain ATCC11845 were treated with a sub-inhibitory concentration of glutaraldehyde [0.01% (vol/vol) and 0.005% (vol/vol) for strain CH-1 and strain ATCC11845, respectively] and assayed for *lptD* expression by qRT-PCR. The results showed that *lptD* transcription increased 3- and 10-fold in strain CH-1 and strain ATCC11845 after incubation with glutaraldehyde, respectively (Figure 2), indicating that *lptD* transcription is induced by glutaraldehyde in both of these strains.

### Low *lptD* Expression Affects *R. anatipestifer* ATCC11845 Growth in TSB Medium

**FIGURE 3:**
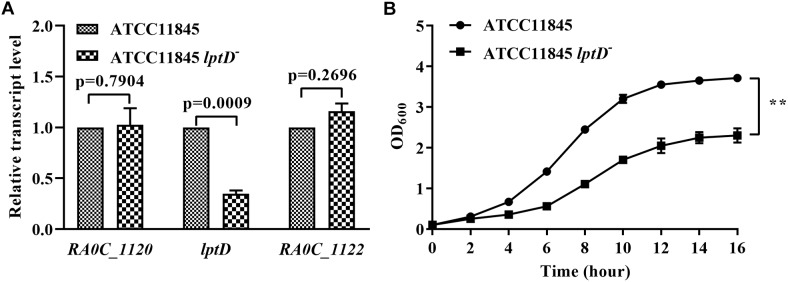
Effect of low *lptD* expression on the growth of *R. anatipestifer* ATCC11845 in TSB. **(A)** The transcription of *RA0C_1120*, *lptD* and *RA0C_1122* in strain ATCC11845 and ATCC11845 *lptD*^−^. qRT-PCR analysis of the transcription of *RA0C_1120*, *lptD* and *RA0C_1122* in ATCC11845 and ATCC11845 *lptD*^−^ in TSB. The data were analyzed using a *t*-test. The error bars represent the standard deviations of three independent experiments. **(B)** The growth of strain ATCC11845 and ATCC11845 *lptD*^−^ in TSB. Cells were grown in 20 ml of TSB medium at 37°C with an initial OD_600_ of 0.05. The OD_600_ values were subsequently measured every 2 h for 16 h, ^∗∗^, *p* < 0.01.

To elucidate the function of *lptD* in *R. anatipestifer*, we attempted to construct an *lptD* mutant strain; however, this effort failed despite numerous attempts, suggesting that *lptD* is an essential gene in *R. anatipestifer*. This result was not unexpected, as *lptD* has been consistently shown to be essential in *E. coli* (Sampson et al., 1989; Braun and Silhavy, 2002; Chng et al., 2010a) and *Salmonella typhimurium* (Dong et al., 2014; Gu et al., 2015). Subsequently, we inserted an erythromycin resistance gene driven by its native promoter upstream of the *lptD* start codon region to decrease *lptD* transcription, which was shown 2-fold lower than in the wild-type strain by qRT-PCR (Figure 3A). The strain with low *lptD* expression was named strain ATCC11845 *lptD*^−^. Moreover, strain ATCC11845 *lptD*^−^ had no significant effect on the transcription of upstream gene *RA0C_1120* and downstream gene *RA0C_1122*, suggesting that it did not cause polar effect to *RA0C_1122* (Figure 3A). Later, strain ATCC11845 *lptD*^−^ was used to evaluate the effect of *lptD* on the growth of strain ATCC11845. The results showed that wild-type strain ATCC11845 grew well in TSB liquid medium, whereas that of strain ATCC11845 *lptD*^−^ was severely impaired (Figure 3B).

### *R. anatipestifer* ATCC11845 *lptD*^−^ Is More Susceptible to Glutaraldehyde and Several Antibiotics Than the Wild-Type Strain

To directly assess whether *lptD* affects the tolerance of *R. anatipestifer* to glutaraldehyde and several antibiotics, including novobiocin, imipenem, rifampicin and polymyxin B, the MICs of glutaraldehyde and antibiotics for strain ATCC11845 and strain ATCC11845 *lptD*^−^ were determined. As shown in Table 2, strain ATCC11845 *lptD*^−^ was more susceptible to glutaraldehyde than strain ATCC11845, with MICs of 0.0125% (vol/vol) and 0.05% (vol/vol) observed for these strains, respectively. The MICs of novobiocin, imipenem, rifampicin and polymyxin B for strain ATCC11845 *lptD*^−^ were 0.025, 0.125, 0.005, and 250 μg/ml, respectively, whereas the MICs for these antibiotics for strain ATCC11845 were 0.05, 0.5, 0.025, and >500 μg/ml, respectively. These results suggested that the *lptD*^−^ strain was more susceptible than the wild-type strain to glutaraldehyde, novobiocin, imipenem, rifampicin and polymyxin B. However, compared to that of ATCC11845, the sensitivity of ATCC11845 *lptD*^−^ to SDS did not have significant change (Table 2).

### Decreased *lptD* Expression Affects *R. anatipestifer* Biofilm Formation and Resistance to Duck Serum

**FIGURE 4:**
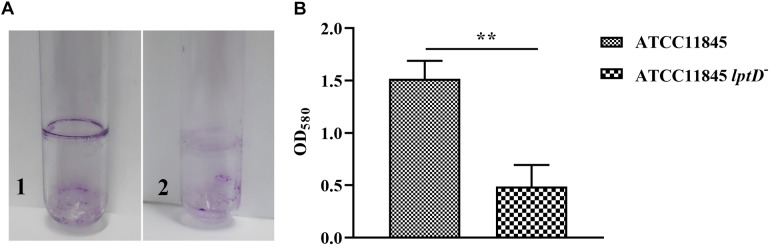
Biofilm formation assay for *R. anatipestifer* ATCC11845 and ATCC11845 *lptD*^−^. **(A)** Representative images of the results of the crystal violet biofilm formation assay. Strain ATCC11845 (1) and ATCC11845 *lptD*^−^ (2) were collected from LB agar plates supplemented with 5% sheep blood and resuspended in TSB supplemented with 5% serum at an OD_600_ of 0.1 in glass tubes and incubated at 37°C without shaking for 24 h. Subsequently, the tubes were washed three times with PBS andstained with 6 ml of 0.1% crystal violet for 30 min at room temperature after carefully removing the bacteria. **(B)** The absorbance of crystal violet-stained biofilm at OD_580_, ^∗∗^, *p* < 0.01.

**FIGURE 5:**
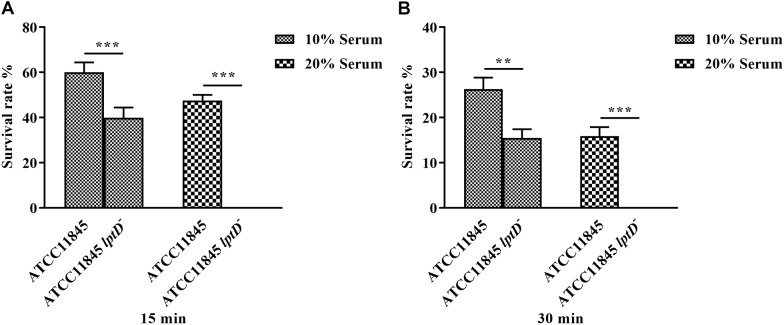
Serum bactericidal assay. Bacteria were incubated with 10 and 20% normal duck serum at 37°C and enumerated after 15 min **(A)** and 30 min **(B)** of incubation. The resistance of strain ATCC11845 *lptD*^−^ to duck serum was significantly reduced compared with that of strain ATCC11845 (^∗∗^, *p* < 0.01, ^∗∗∗^, *p* < 0.001).The survival rate (%) was calculated as follows: (bacterial CFU with serum treatment/bacterial CFU with PBS treatment) × 100.

Previous studies showed that LPS is a primary component of biofilms (Murphy et al., 2014; Alshalchi and Anderson, 2015). Thus, the role of *lptD* in *R. anatipestifer* biofilm formation was examined in test tubes. The results showed that strain ATCC11845 *lptD*^−^ was significantly attenuated in biofilm formation compared to the wild-type strain (Figure 4A). The OD_580_ values for strain ATCC11845 and strain ATCC11845 *lptD*^−^ were 1.52 and 0.48, respectively, suggesting that the biofilm formation of strainATCC11845 *lptD*^−^ was significantly lower than that of the wild-type strain (Figure 4B). These results indicated that the decreased expression of *lptD* had an effect on *R. anatipestifer* biofilm formation. Next, a bactericidal assay was performed to determine whether *lptD* is involved in the resistance of *R. anatipestifer* to duck serum. As shown in Figure 5, the survival rates of strain ATCC11845 and strain ATCC11845 *lptD*^−^ in 10% non-inactivated serum for 15 min were 60.1 and 39.9%, respectively. In contrast, when incubated in 20% non-inactivated serum for 15 min, all strain ATCC11845 *lptD*^−^ bacteria were killed, whereas the survival rate of strain ATCC11845 was 47.6%. When incubated in 10% non-inactivated serum for 30 min, the bacterial survival rates of strain ATCC11845 and strain ATCC11845 *lptD*^−^ were 26.3 and 15.5%, respectively. When the concentration of non-inactivated serum was increased to 20% for 30 min, all strain ATCC11845 *lptD*^−^ cells were killed, whereas the survival rate of strain ATCC11845 was 15.9%. Taken together, these results suggested that the decreased expression of *lptD* in strain ATCC11845 *lptD*^−^ resulted in significantly greater sensitivity to duck serum than the wild-type strain.

### Decreased *lptD* Expression Affects the Colonization of *R. anatipestifer* ATCC11845 *in vivo*

**FIGURE 6:**
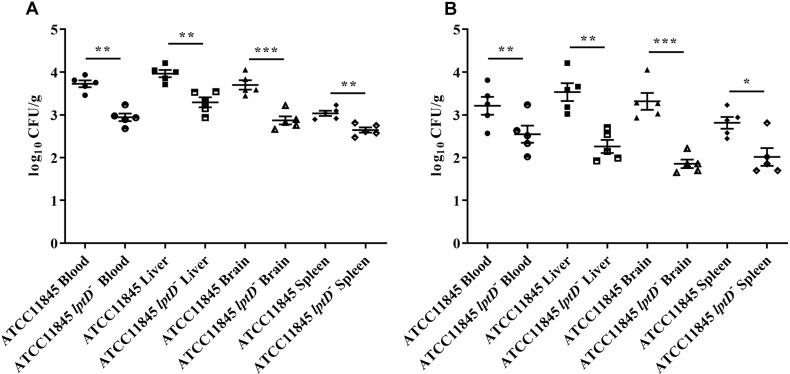
Colonization of *R. anatipestifer* ATCC11845 and ATCC11845 *lptD*^−^ *in vivo*. The strain ATCC11845 (10^9^ CFU) and ATCC11845 *lptD*^−^ (10^9^ CFU) strains were injected into the leg muscles of 3-day-old ducklings. At 12 h **(A)** and 18 h **(B)** postinfection, bacteria were isolated from the livers, brains, spleens, and blood according to the methods described in the “Materials and Methods” section. The data points represent the log_10_CFU/g of individual animals in the indicated organs, with the bars representing the median values (*n* = 5), ^∗^, *p* < 0.05, ^∗∗^, *p* < 0.01, and ^∗∗∗^, *p* < 0.001.

To further investigate whether *lptD* contributes to the colonization dynamics of *R. anatipestifer* during systemic infection, colonization assay was conducted by infecting 3-day-old ducklings with strain ATCC11845 or strain ATCC11845 *lptD*^−^ by leg muscle injection. Compared to ducklings infected with strain ATCC11845, at 12 h postinoculation, a notable reduction in the bacterial load was observed in ducklings infected with strain ATCC11845 *lptD*^−^ in the heart blood (6-fold reduction), liver (4-fold reduction), brain tissue (7-fold reduction) and spleen (2-fold reduction) (Figure 6A). At 18 h postinoculation, compared to strain ATCC11845, significant reductions in the strain ATCC11845 *lptD*^−^ bacterial loads were still observed in the blood (4-fold reduction), livers (23-fold reduced), brains (42-fold reduction) and spleens (4-fold reduction) (Figure 6B). These results suggest that *lptD* is involved in the pathogenesis of *R. anatipestifer*.

## Discussion

With the increasing use of antimicrobials, resistance in *R. anatipestifer* is becoming an important concern. Compared to strain ATCC11845, strain CH-1 is resistant to many antibiotics. At present, it is unknown the resistance to organic solvents in strain CH-1 and strain ATCC11845. Hydrophobic organic solvents are extremely toxic to microorganisms, even at the very low concentration of 0.1% (vol/vol). The first reported organic-solvent-tolerant bacterium was *Pseudomonas* (Inoue and Horikoshi, 1989). As an organic solvent, glutaraldehyde has been used extensively as a hydrophobic drug. The MIC of glutaraldehyde for strain CH-1 and strain ATCC11845 was assessed to determine if the resistance of these strains to this disinfectant is different. We observed that strain CH-1 and strain ATCC11845 differ in their tolerance to glutaraldehyde, with strain ATCC11845 being more susceptible than strain CH-1.

It was previously reported that *imp*/*ostA* (namely, *lptD*) is involved in glutaraldehyde resistance in a clinical strain of *H. pylori* (Chiu et al., 2007, 2009). To investigate whether the difference in glutaraldehyde resistance of *R. anatipestifer* CH-1 and ATCC11845 is associated with *lptD*, the transcription of this gene was measured in these strains by qRT-PCR. The result showed that the level of *lptD* transcription in *R. anatipestifer* CH-1 was 2-fold higher than that observed in strain ATCC11845. After that, when *R. anatipestifer* was treated with a subinhibitory concentration of glutaraldehyde [0.01% (vol/vol) for strain CH-1, 0.005% (vol/vol) for strain ATCC11845], the transcription levels of *lptD* were both increased. Altogether, these results suggested that the transcription level of *lptD* was associated with glutaraldehyde resistance, which is consistent with the results reported for *H. pylori* (Chiu et al., 2009).

The *lpt* genes are widely distributed in bacteria that do or do not produce LPS (Putker et al., 2015). LPS transport has been studied extensively in the β- and γ-proteobacteria *N. meningitides* and *E. coli*, respectively. Seven Lpt proteins have been shown to be involved in this process. Searching the genome of *R. anatipestifer* ATCC11845 for homologs of Lpt proteins revealed that LptA, LptB, LptD, LptF, and LptG (RA0C_1913, RA0C_1993, RA0C_1121, RA0C_0335, and RA0C_1496) exhibit 42, 53.78, 48, 19.96, and 30.56% identity to previously identified proteins in *E. coli*, respectively. Homologs of LptC and LptE were not identified through sequence analysis of the *R. anatipestifer* ATCC11845 genome. In *E. coli*, LptD forms a complex with lipoprotein LptE to help LPS transport across outer membrane (Chimalakonda et al., 2011). Overall, it appears that *R. anatipestifer* transports LPS to the outer membrane via similar Lpt machinery, but there are some differences in the process due to a lack of LptC and LptE. LPS is essential in most gram-negative bacteria, with the notable exception of *N. meningitides* (Steeghs et al., 1998). The *lptD* gene has been consistently shown to be an essential gene in *E. coli* (Sampson et al., 1989; Braun and Silhavy, 2002; Chng et al., 2010a) and *S. typhimurium* (Putker et al., 2015). Several attempts were made to generate an *lptD* knockout in *R. anatipestifer* to study the function of this gene directly. However, the failure to obtain this mutant suggested that *lptD* is also an essential gene in *R. anatipestifer*. Subsequently, a *R. anatipestifer* ATCC11845 *lptD*^−^ strain was constructed with low *lptD* expression, which was confirmed by qRT-PCR. The results of the glutaraldehyde sensitivity assay showed that *R. anatipestifer* ATCC11845 *lptD*^−^ was more susceptible to glutaraldehyde than strain ATCC11845, suggesting that LPS forms a barrier that protects cells from glutaraldehyde and promotes resistance. Although we constructed a complemented strain using the shuttle plasmid pLMF03, the wild-type phenotype was not restored in this strain. The lack of complementation could be due to problems related with the backbone vector or *lptD* expression levels. Thus, it was absolutely required to establish a method for conditional mutant to study the function of the essential gene in *R. anatipestifer*. Furthermore, several attempts to extract LPS from strain ATCC11845 and strain ATCC11845 *lptD*^−^ failed. In addition, we have analyzed the genetic organization of the *lptD* in the genome of *R. anatipestifer* ATCC11845. As shown in Supplementary Figure S2, it was shown that *lptD* (*RA0C_1121*) was not located in an operon, however, *RA0C_1122* and *RA0C_1123* formed an operon through bioinformatic analysis. The direction of transcription of *lptD* locus and gene *RA0C_1120* is reversed. The intergenic region between *lptD* and *RA0C_1120* or *RA0C_1122* is 22 bp and 89 bp, respectively. qRT-PCR revealed that strain ATCC11845 *lptD*^−^ had no significant effect on the transcription level of *RA0C_1120* and *RA0C_1122*.

Lipopolysaccharide is a primary component of biofilm in *P. aeruginosa* (Murphy et al., 2014; Alshalchi and Anderson, 2015). To investigate whether *lptD* affects biofilm formation, the biofilms of *R. anatipestifer* ATCC11845 and strain ATCC11845 *lptD*^−^ were examined. The results showed that biofilms formed by strain ATCC11845 *lptD*^−^ had decreased biomass compared to those formed by the wild-type strain. Serum bactericidal assays showed that strain ATCC11845 *lptD*^−^ was significantly more sensitive to duck serum than the wild-type strain. *In vivo*, the bacterial loads of *R. anatipestifer* ATCC11845 *lptD*^−^ were lower than those of the wild-type strain in the blood, livers, brains and spleens of ducklings. Taken together, these results suggested that *lptD* is involved in glutaraldehyde resistance and bacterial virulence in *R. anatipestifer*.

## Data Availability

The raw data supporting the conclusion of this manuscript will be made available by the authors, without undue reservation, to any qualified researcher.

## Author Contributions

ML, AC, and FB conceived and designed the experiments. LH and TM constructed the RA ATCC11845 strain with low *lptD* expression and assessed the sensitivity of tested strains to glutaraldehyde and SDS. DZ, MW, and YL performed the qRT-PCR to determine the level of *lptD* transcription. LZ, XC, YY, and JH performed the biofilm formation assay and animal experiments. LP, MR, MW, RJ, SC, and XZ analyzed the data. BT, YW, QY, and SZ contributed to the reagents, materials, and analysis tools. LH, ML, FB, and AC wrote the manuscript. All authors reviewed the manuscript.

## Conflict of Interest Statement

The authors declare that the research was conducted in the absence of any commercial or financial relationships that could be construed as a potential conflict of interest.
