# Supplementary material for: Role of LptD in Resistance to Glutaraldehyde and Pathogenicity in Riemerella anatipestifer
Source: Front Microbiol. 2019 Jun 21;10:1443. doi: 10.3389/fmicb.2019.01443 (PMC6598057; doi:10.3389/fmicb.2019.01443)
Supplement: Supplementary file 1 [file Image_1.pdf]

Supplementary Material

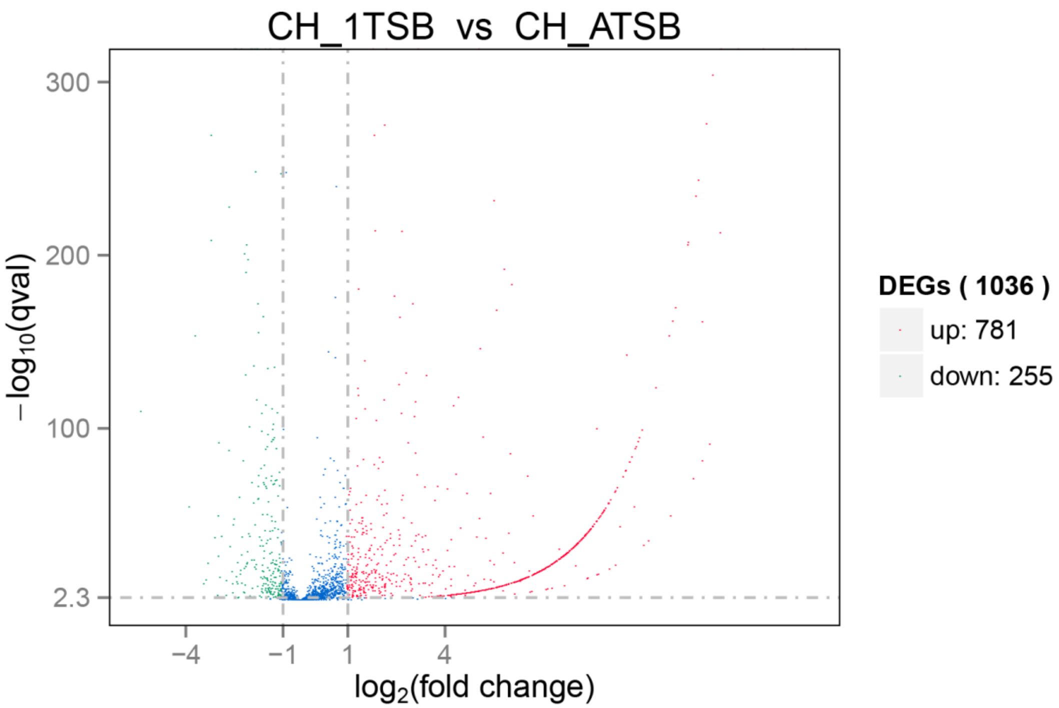

Supplementary Figure S1 Differential gene transcription of *R. anatipestifer* CH-1 (CH_1TSB) compared to strain ATCC11845 (CH_ATSB) in TSB. The x-axis of the chart shows $\log_2$-based fold changes of transcripts in cells grown in iron-limited medium or TSB medium. The y-axis of the chart shows the statistical significance. Each dot in the chart represents one annotated gene. Red dots: upregulated and green dots: downregulated.
