# Supplementary material for: Role of LptD in Resistance to Glutaraldehyde and Pathogenicity in Riemerella anatipestifer
Source: Front Microbiol. 2019 Jun 21;10:1443. doi: 10.3389/fmicb.2019.01443 (PMC6598057; doi:10.3389/fmicb.2019.01443)
Supplement: Supplementary file 2 [file Image_2.pdf]

Supplementary Material

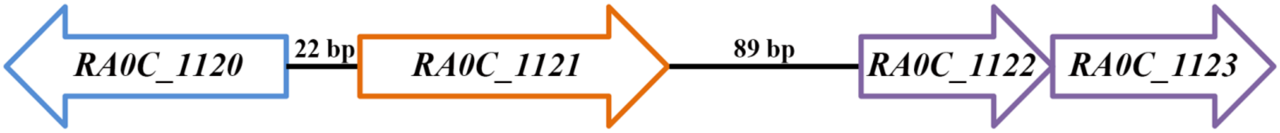

Supplementary Figure S2 The genetics organization of *lptD* in the genome RA ATCC11845 through bioinformatic analysis. The same color boxes represent an operon. *RA0C_1121* (*lptD*) was not located in an operon. *RA0C_1122* and *RA0C_1123* formed an operon. The direction of transcription of *lptD* locus and gene *RA0C_1120* is reversed. The intergenic region between *lptD* and *RA0C_1120* or *RA0C_1122* is 22 bp and 89 bp, respectively.
